# Distribution and Abundance of *Aedes aegypti* and *Aedes albopictus* (Diptera: Culicidae) in Benin, West Africa

**DOI:** 10.3390/tropicalmed8090439

**Published:** 2023-09-07

**Authors:** Germain Gil Padonou, Alphonse Keller Konkon, Albert Sourou Salako, David Mahouton Zoungbédji, Razaki Ossè, Arthur Sovi, Roseric Azondekon, Aboubakar Sidick, Juvénal Minassou Ahouandjinou, Constantin Jesukèdè Adoha, André Aimé Sominahouin, Filémon Tatchémè Tokponnon, Bruno Akinro, Haziz Sina, Lamine Baba-Moussa, Martin Codjo Akogbéto

**Affiliations:** 1 Centre de Recherche Entomologique de Cotonou (CREC), Cotonou 06 BP 2604, Benin; konkonkelleralphonse@gmail.com (A.K.K.); albertsourousalako@yahoo.fr (A.S.S.); davidzoungbedji91@gmail.com (D.M.Z.); aosse@gouv.bj (R.O.); roseric_2000@yahoo.fr (R.A.); sidick_aboubakar@yahoo.fr (A.S.); juveminassou@gmail.com (J.M.A.); adohaj.constantin@yahoo.fr (C.J.A.); andrsominahouin@yahoo.fr (A.A.S.); filemont@yahoo.fr (F.T.T.); akinrobruno@gmail.com (B.A.); akogbetom@yahoo.fr (M.C.A.); 2 Laboratory of Biology and Molecular Typing in Microbiology, Department of Biochemistry and Cellular Biology, Faculty of Sciences and Techniques, University of Abomey-Calavi, Cotonou 05 BP 1604, Benin; sina.haziz@gmail.com (H.S.); laminesaid@yahoo.fr (L.B.-M.); 3 École de Gestion et d’Exploitation des Systèmes d’Élevage, Université Nationale d’Agriculture de Porto-Novo, Porto-Novo 01 BP 55, Benin; 4 Faculty of Agronomy, University of Parakou, Parakou BP 123, Benin; 5 Faculty of Infectious and Tropical Diseases, Disease Control Department, The London School of Hygiene & Tropical Medicine, Keppel Street, London WC1E 7HT, UK

**Keywords:** *Aedes aegypti*, *Aedes albopictus*, distribution, abundance, biting behaviour, Benin

## Abstract

Updated information on the distribution and abundance of *Aedes aegypti* and *Aedes albopictus* is crucial to prepare African countries, such as Benin, for possible arboviral disease outbreaks. This study aims to evaluate the geographical distribution, abundance and biting behaviour of these two vectors in Benin. Three sampling techniques were used in this study. The collection of *Aedes* spp. adults were made through human landing catch (HLC), immatures were captured with the use of ovitraps, and a dipping technique was used for the collection of *Aedes* spp. in 23 communes located along the North–South and East–West transect of Benin. Adult *Aedes* mosquitoes were collected indoors and outdoors using HLC. Mosquito eggs, larvae and pupae were collected from containers and ovitraps. The adult mosquitoes were morphologically identified, then confirmed using a polymerase chain reaction (PCR). Overall, 12,424 adult specimens of *Aedes* spp. were collected, out of which 76.53% (n = 9508) and 19.32% (n = 2400) were morphologically identified as *Ae. aegypti* and *Ae. albopictus*, respectively. Geographically, *Ae. aegypti* was found across the North–South transect unlike *Ae. albopictus*, which was only encountered in the southern part of the country, with a great preponderance in Avrankou. Furthermore, an exophagic behaviour was observed in both vectors. This updated distribution of *Aedes* mosquito species in Benin will help to accurately identify areas that are at risk of arboviral diseases and better plan for future vector control interventions.

## 1. Background

The *Aedes* (Stegomyia) *albopictus* (Skuse, 1894) tiger mosquito is currently one of the most invasive species that has successfully colonised most tropical and temperate regions globally [1]. Native to Southeast Asia, this species has spread to several other parts of the world except for Antarctica [1,2]. Genetic evidence suggests that this global invasion of *Ae. albopictus* is strongly associated with anthropogenic actions, such as the trade of used tires (passive dispersal), transport (sea and land) and travel [3,4,5]. Additionally, Kamgang et al. [6] suggest that the ecological plasticity of the *Aedes* species allows them to proliferate in a wide range of climates and habitats. Given the ability of *Ae. albopictus* to tolerate low temperatures [7], it is now present in temperate regions where it lives in sympatry with *Ae. aegypti*. Both species were reported to share the same larval habitats in urban and peri-urban environments [8]. Such larval habitats can be domestic (water reservoirs and flowerpots), peri-domestic (discarded reservoirs and used tires) or natural breeding sites (tree holes and plant axils) [6,9]. The ease of expansion for *Ae. aegypti* and *Ae. albopictus* has created conducive conditions for the emergence of human arboviral diseases, such as Chikungunya, Zika, dengue fever and yellow fever, in new geographical areas [10]. Over the past 30 years, the distribution and epidemiological impact of these arboviral diseases on public health have increased considerably [11]. In Africa, *Ae. albopictus* was first identified in South Africa in 1989 and was quickly brought under control [12]. Two years later (1991), in West Africa, this vector was reported in the Delta State in Nigeria where it has since spread [4,13]. In Central Africa, *Ae. albopictus* was reported in Cameroon in 2000 [14] and has since expanded across the region. Curiously, this expansion of *Ae. albopictus* in Central Africa coincided with the emergence of dengue (denv), zika (zikv) and chikungunya (chikv) viruses in urban settings [15]. Paupy et al. [16] suspected that this mosquito would play a leading role in the occurrence of these diseases in the region. In West Africa, although the first detection of *Ae. albopictus* was reported in 1991 in Nigeria, there was no report of this *Aedes* species in neighbouring Benin until 2021, a country that shares 773 kilometres of border with Nigeria [17]. Between July and August 2010, two cases of dengue were diagnosed in France in travellers from Cotonou, Benin [18,19]. Between April and July 2019, eight confirmed cases of dengue, including two deaths were recorded in the departments of Atlantique, Littoral and Ouémé in southern Benin [20]. According to Padonou et al. [21], the diagnosis of confirmed dengue cases in a given area is an indication of the area’s strong infestation by mosquitoes of the *Aedes* genus. Cases of yellow fever were reported in Nigeria, a country neighboring Benin [22]. In addition, the high frequency of people travelling between the two countries increases the risk of yellow fever in Benin.

Recently, the dengue serotype 3 was detected in *Aedes* vector populations in Benin [23]. Moreover, cases of dengue virus infections have been recorded in Rosso area in Senegal [24,25]. In 2022, dengue epidemics affected the economic capital of Côte d’Ivoire, with 380 detected cases [25]. In addition, several studies showed a strong association between the distribution of the vectors and the risk of occurrence of a disease [21,26]. In the case of arboviral diseases, the obtained results have enabled the development of risk maps [27,28,29]. These maps are useful for decision-making, as they allow for control interventions to target with precision the most affected areas. In Benin, the distribution area of *Ae. albopictus* is still poorly known. Moreover, even though *Ae. aegypti* is very often found in routine entomological collections, an update of its distribution area in the country is required. This study established the distribution map for *Ae. aegypti* and *Ae. albopictus* in Benin. This proposed tool is a prerequisite that will be used to prepare the country’s response to potential arboviral diseases epidemics.

## 2. Methods

### 2.1. Study Area

**Figure 1:**
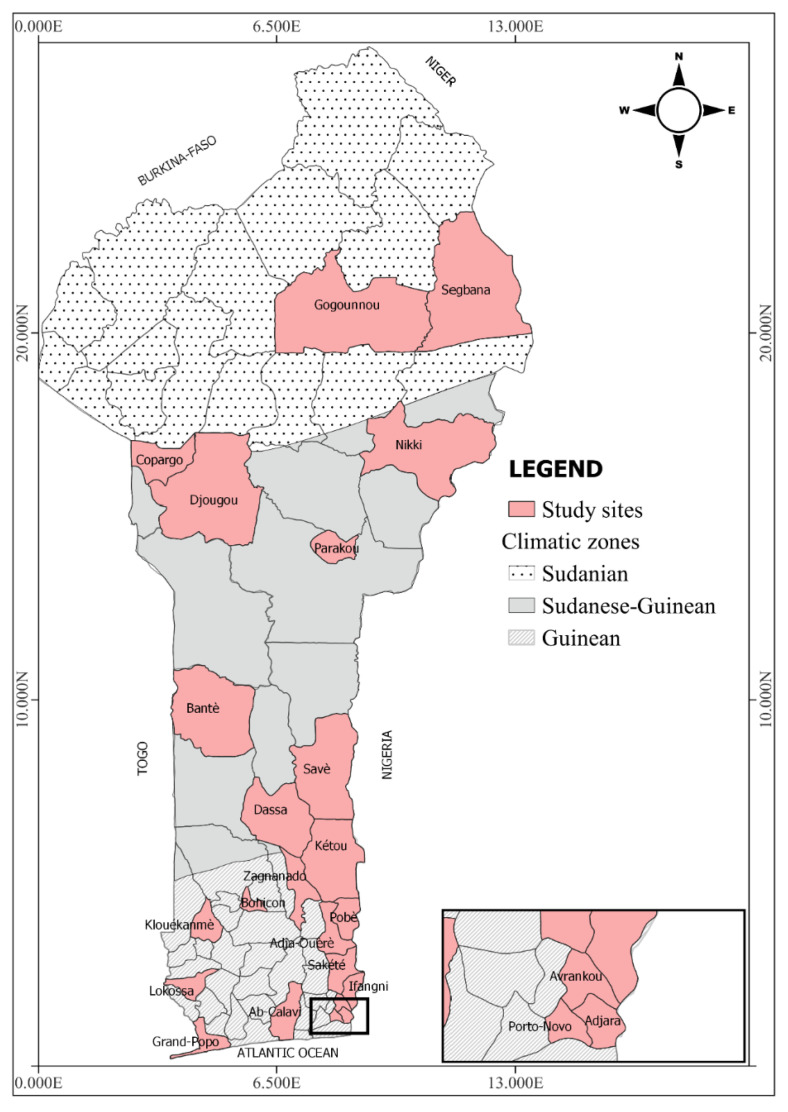
Map showing the study area (the map was drawn using the QGIS 3.32.2 Lima Software).

This study was carried out in Benin, more specifically in 23 of the 77 communes in the country (Figure 1). Among the 23 investigated communes, ten were selected due to their closeness to Nigeria, where *Ae. albopictus* was first detected three decades ago. The communes were classified into three zones (urban, peri-urban and rural), according to their degree of urbanization, presence of vegetation, population density, presence of modern infrastructure and type of houses.

The 23 communes were selected according to their representativeness of the three major eco-climatic zones in the country [30].

#### 2.1.1. Area of Degraded Forests with a Subequatorial Climate

This area is characterised by two rainy seasons (mid-March to mid-July and September to November) and two dry seasons (December to mid-March and mid-July to August). Its annual rainfall varies from 1300 to 1500 mm per year [31]. The communes surveyed in this area included Abomey-Calavi, Porto-Novo, Adjara, Avrankou, Ifangni, Sakété, Pobè, Kétou, Lokossa, Klouékanmè, Zagnanado, Bohicon and Grand Popo (Figure 1). Abomey-Calavi and Porto-Novo are the most urbanized while the others are peri-urban or rural.

#### 2.1.2. Area of Savannas with a Sudano-Guinean Climate

This area has two seasons: a rainy season (April to October) and a dry season (November to March). Its annual rainfall varies between 1200 and 1300 mm [31]. The communes investigated in this area were urban (Parakou), peri-urban (Djougou and Dassa) and rural (Savè, Bantè, Nikki and Corpago) (Figure 1).

#### 2.1.3. Area of Savannahs with a Sudanian Climate

A dry season from November to May and a rainy season from June to October characterise this area. Its climate is of the Sudanian type. The rural communes of Gogounou and Ségbana were investigated here [31] (Figure 1).

Overall, in each of these communes, the surveys were carried out in two villages that were selected at random. The geographical coordinates of the surveyed villages were recorded using a Global Positioning System (GPS) in order to map the distribution and density of *Ae. albopictus* and *Ae. aegypti*.

### 2.2. Mosquito Collection Techniques

To determine the different mosquito species of the *Aedes* genus, even those present at low frequencies in all the study communes, three sampling techniques were used between July 2021 and October 2022. These included the following.

#### 2.2.1. Collection of Mosquito Immature Stages

Larvae and pupae were sampled both indoors and outdoors in different types of larval habitats, including the following.

- Domestic containers: flowerpots, drums, cups, water storage containers (cement-made cisterns and earthen jars), buckets, garbage cans, pet water bowls, bottles, mortars and barrels.
- Discarded containers: plastic bags, abandoned machinery (refrigerators, freezers) and tin cans.
- Tires
- Natural breeding sites: fruit shells, plant leaf axils, coconut and tree and root holes.
- Others: wheelbarrows, abandoned canoes and cars, and pipes in the ground.

These immature stages were then transported to the CREC where they were reared at insectary conditions (temperature: 28 ± 1 °C, relative humidity: 70–80%, 14 h: 10 h light: dark photoperiod) until adulthood.

#### 2.2.2. Using the Ovitrap Method

The ovitraps used in this study were made from a painted black polyethylene container that contained 50 mL of water. A rectangular plywood support (5 cm × 20 cm) was introduced to the black polyethylene container to serve as a support for laying mosquito eggs. Twelve ovitraps were placed per site, with four sites surveyed per commune. These traps were approximately 100 metres apart from each other. They were fixed to a tree or a wall approx. 1.5 cm from the ground using a nail and a metal string for approx. 5 to 7 days in the domestic (yard) and peri-domestic environments. The ovitraps were regularly inspected to avoid egg hatching as much as possible. However, when the larvae were observed in the traps, they were collected and brought back to the insectary of the “Centre de Recherche Entomologique de Cotonou” (CREC) for rearing until adulthood. The adults were then identified, counted and released into cages. Between the fifth and seventh day, the ovitraps were withdrawn and the hardboard plate was brought back to the insectary of the CREC. The eggs that were laid were counted and put in water at the insectary to enable hatching and development until adulthood. The emerging adults were put into a refrigerator (−20 °C) for 15 min to immobilise them before they were identified. They were grouped into a pool of 10 individuals, taking into account their geographical origin, sex and date of collection, and then stored at −80 °C.

#### 2.2.3. Human Landing Catch (HLC)

This method, which collects adult mosquitoes, was used in two sites (one central and one peripheral) that were selected in each commune. In each site, the collections using human bait were carried out from 7 a.m. to 6 p.m. in two houses, with one collector seated inside and a second outside each house, which resulted in a total of four collectors/site/day and 16 collectors/commune/day. Indeed, two teams of eight collectors each were, therefore, formed per commune. The first team collected samples from 7 a.m. to 1 p.m. and was replaced by the second team from 1 p.m. to 6 p.m. The collectors used haemolysis tubes to capture the mosquitoes that tended to bite their bare feet and legs.

### 2.3. Morphological and Molecular Identification

The adult mosquitoes (males and females) from the three sampling methods (ovitrap method, collection of immature stages and HLC) were morphologically identified using a binocular microscope and the taxonomic keys from Edwards [32] and Yiau-Min [33]. The specimens of *Aedes* spp. were referenced, preserved on RNA Later and grouped according to their species, locality, date and location (indoor/outdoor).

The legs of the specimens of *Ae. albopictus* were used for the extraction of their DNA using the protocol of Linton et al. [34]. Due to a high degree of interspecific variation [35,36], the ITS2 nuclear ribosomal spacer gene was amplified by a PCR using the primers 5.8 S and 28 S [37,38]. The PCR product of *Ae. albopictus* was 509 bp and 518 bp according to the sequences published on GenBank M95127 [38] and L22060 [39], respectively. The PCR was performed in a volume of 50 μL containing a 1× PCR buffer, 2 mM of MgCl_2_, 0.2 μM of each dNTP, 100 pM of each primer, 2 U of Taq DNA polymerase and 2 μL of DNA to be amplified. *Aedes aegypti* was used as the negative control. The amplification was carried out using a thermocycler according to the following programme: an initial denaturation at 94 °C for 10 min; 40 cycles of denaturation at 94 °C for 1 min; an initial hybridization at 50 °C for 1 min and hybridization at 72 °C for 1 min. The final hybridization was performed at 72 °C for 10 min. The products were migrated onto 1.5% agarose gels containing 0.5 μg/mL of ethidium bromide. The gels were photographed using a polaroid camera under UV illumination following standard procedures [40].

### 2.4. Ethical Considerations

The protocol of this study was reviewed and approved by the “Comité Institutionnel d’Ethique pour la Recherche en Santé du Centre de Recherche Entomologique de Cotonou” (CIERS-CREC) (Ethical approval N°06-22/CREC/CIERS-CREC /SG). The collectors were selected from the different study sites and trained to collect mosquitoes before they were bitten. They were all vaccinated against yellow fever and subjected to regular check-ups at the nearest health facility. In the occurrence of fever, they were immediately taken care of.

### 2.5. Data Analysis

The data were analysed using the R statistical software, version 4.1.2 [41]. The Chi-square test of the comparison of the proportions was used to compare the distribution of each species according to the different eco-climatic areas. The species richness (S), which corresponded to the number of collected species, and their relative abundance (Pi) were computed (Pi = ni/N, where ni = number of species i; N = total number of species encountered; i = 1:S) per study site.

The Shannon–Weaver index (H), which showed the diversity of the species, was determined for all 23 sites according to the following formula: $H=-\sum_{i=1}^{S}P_{i}\times {log}_{2}{(P}_{i})$ [42].

The equitability index was also calculated as $\;E=\frac{\;H}{\log_{2}S}$ [43].

All these parameters were determined using the combined number of *Aedes* species obtained with the three sampling techniques to assess the proportion of the main dengue vectors and estimate the level of risk.

The higher or lower biting risk for each collector from each mosquito species (*Ae. aegypti* or *Ae. albopictus*) was calculated as the number of mosquito species collected divided by the number of collectors per day. The Poisson method was used to estimate the confidence intervals of the HBRs and compare them between the study sites and locations (indoors and outdoors). The risk of receiving greater or fewer bite(s) from each mosquito species (*Ae. aegypti* or *Ae. albopictus*) indoors compared to outdoors was assessed by calculating the rate ratio (RR). A difference was considered significant when the p-value was less than 0.05.

## 3. Results

### 3.1. Diversity of the Aedes Mosquito Species in Benin

A total of 12,424 specimens of *Aedes* spp. were collected over a two-year period using the three sampling methods. The predominant species were *Ae. aegypti* (76.53% [75.77–77.27], n = 9508) and *Ae. albopictus* (19.32% [18.63–20.02], n = 2400). The other *Aedes* species included *Ae. luteocephalus* (3.50% [3.18–3.84]; n = 435), *Ae.* (*Neomelaniconion*) *palpalis* (0.45% [0.34–0.58]; n = 56), *Ae. vittatus* (0.13% [0.07–0.21]; n = 16) and *Ae. africanus* (0.07% [0.03–0.14]; n = 9) (Table 1). The lowest number of *Aedes* were observed in Savè (n = 114) and the highest in Ifangni (n = 4106). The observed species richness was six, four and three, respectively, for the subequatorial, Sudano-Guinean and Sudanian areas (Table 2). According to the equitability index (E), the abundance of species from the *Aedes* genus was low (0.07 [0.03–0.012]) in the Sudanian area, as well as in the Sudano-Guinean area (0.08 [0.05–0.10]). The same applied to the Shannon–Weaver indices, which revealed a low diversity in the northern (H = 0.08 [0.03–0.13]) and central (H = 0.11 [0.08–0.14]) regions of Benin. Meanwhile, the equitability and Shannon–Weaver indices were relatively close to one in southern Benin and were, respectively, E = 0.42 [0.41–43] and H = 0.76 [0.74–0.77].

### 3.2. Distribution and Abundance of Aedes Mosquitoes across the Study Sites

**Figure 2:**
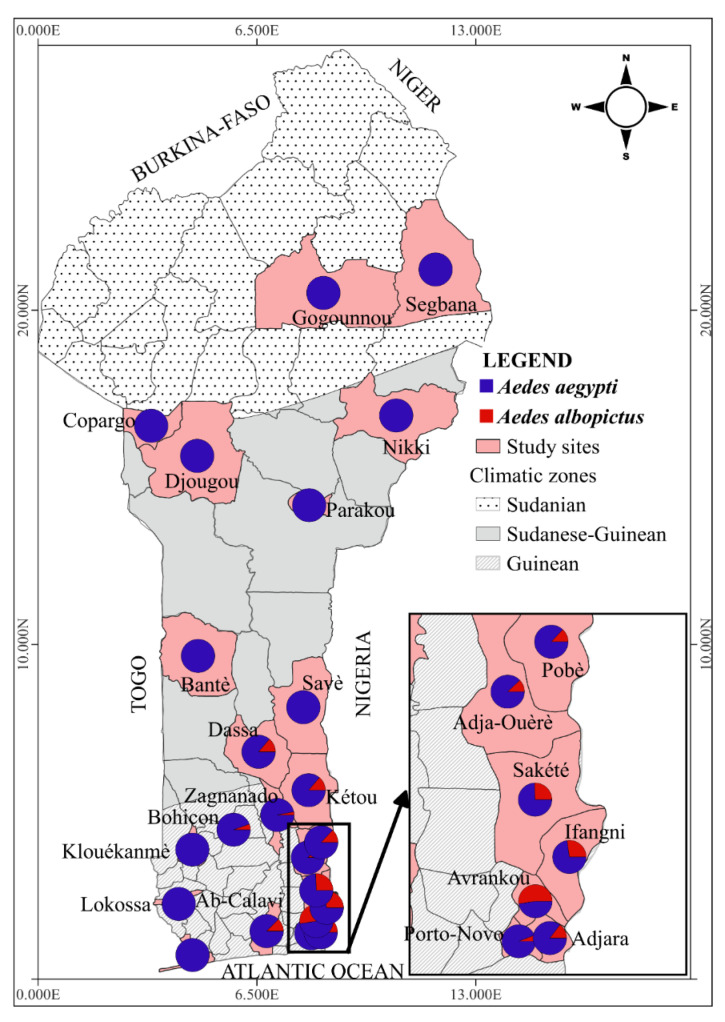
Distribution and relative abundance of *Ae. albopictus* and *Ae. aegypti* on a North–South Benin transect (the map was drawn using the QGIS 3.32.2 Lima Software).

Twenty-three study communes distributed across the three eco-climatic areas were included in this study (Figure 2). *Ae. albopictus* was identified in 12 communes, including 11 (Avrankou, Adjara, Porto-Novo, Abomey-Calavi, Ifangni, Sakété, Pobè, Kétou, Adja-Ouèrè, Bohicon and Zangnanado) in the south of the country where the climate is subequatorial and 01 (Dassa) in the centre of the country with a Sudano-Guinean climate. No specimens of *Ae. albopictus* were observed in the remaining 11 communes.

Specifically, in Southern Benin (subequatorial climate), the following species of *Aedes* spp. were reported: *Ae. aegypti* 71.27% (n = 7116); *Ae. albopictus* 23.71% (n = 2367); *Ae. luteocephalus* 4.34% (n = 433) and other *Aedes* species 0.69% (n = 69) (Table 3). In this climatic zone, *Ae. albopictus* was not collected in three communes (Lokossa, Klouekanmè and Grand-Popo). However, the highest frequency of this mosquito species was recorded in Avrankou, a rural commune (51.72%: n = 196), and the lowest frequencies were recorded in Porto-Novo, an urban commune (7.41%: n = 133), and Zangnanado, a rural commune (3.08%: n = 6) (Table 1).

Among the nine communes surveyed in the Sudano-Guinean and Sudanian areas, *Ae. albopictus* was only collected in Dassa, a peri-urban commune, with a frequency of 1.35% (33/2439) (Table 3). As observed in Southern Benin (subequatorial climate), *Ae. aegypti* was also the predominant *Aedes* species in the northern part of the country where the climate was of the Sudano-Guinean or Sudanian type.

Table 4 shows the *Aedes* species composition and abundance according to the sampling method. Of the 12,424 specimens of *Aedes* spp. that were collected, the abundance and percentage for each was: ovitrap 66.75% (n = 8294); HLC 27.11% (n = 3368) and the collection of immature stages 6.13% (n = 762). *Ae. aegypti (*76.53%, 9508/12,424*) and Ae. albopictus* (19.32%, 2400/12,424) were the most abundant *Aedes* species collected using all three collection methods. Furthermore, the highest *Aedes* species diversity was observed using HLC.

### 3.3. Molecular Identification of Ae. albopictus

A sample of 315 mosquitoes morphologically identified as *Ae. albopictus* were processed using a PCR. All the individuals that were analysed showed a 509 bp fragment, confirming them as *Ae. albopictus* (Table 5).

### 3.4. Human Biting Rate (HBR) in Ae. aegypti and Ae. albopictus

The biting rates of *Ae. aegypti* and *Ae. albopictus* varied significantly from commune to commune, according to the location (indoor or outdoor). Overall, the mean biting rate for the whole study area was 4.31 bites/person/day (b/p/d) [4.02–4.61] for *Ae. aegypti* against 0.95 b/p/d [0.81–1.09] for *Ae. albopictus* (Table 6 and Table 7). The biting rate of *Ae. aegypti* was four times higher than *Ae. albopictus* (*p* < 0.001).

The highest biting rate of *Ae. aegypti* was recorded in July 2021 (rainy season) in Porto-Novo (9.38 b/p/d [7.93–11.00]) and the lowest in Avrankou in August 2022 (0.19 b/p/d [0.038–0.55]) (Table 6). For *Ae. albopictus*, the highest biting rate was observed in August 2022 in Avrankou (2.75 b/p/d [2.00–3.69]) and the lowest in Porto-Novo (0.38 b/p/d [0.14–0.82]) in July 2021 (Table 7).

The data also revealed that the mean outdoor HBR was higher in both species (7.02 b/p/d for *Ae. aegypti* and 1.51 b/p/d for *Ae. albopictus*) than the mean indoor HBR (1.59 b/p/d for *Ae. aegypti* and 0.39 b/p/d for *Ae. albopictus*). Similarly, the rate ratio (RR) < 1 observed for both *Ae. aegypti* and *Ae. albopictus* (Table 6 and Table 7) indicated that the risk for receiving mosquito bites was lower indoors than outdoors.

### 3.5. Type of Breeding Sites

**Figure 3:**
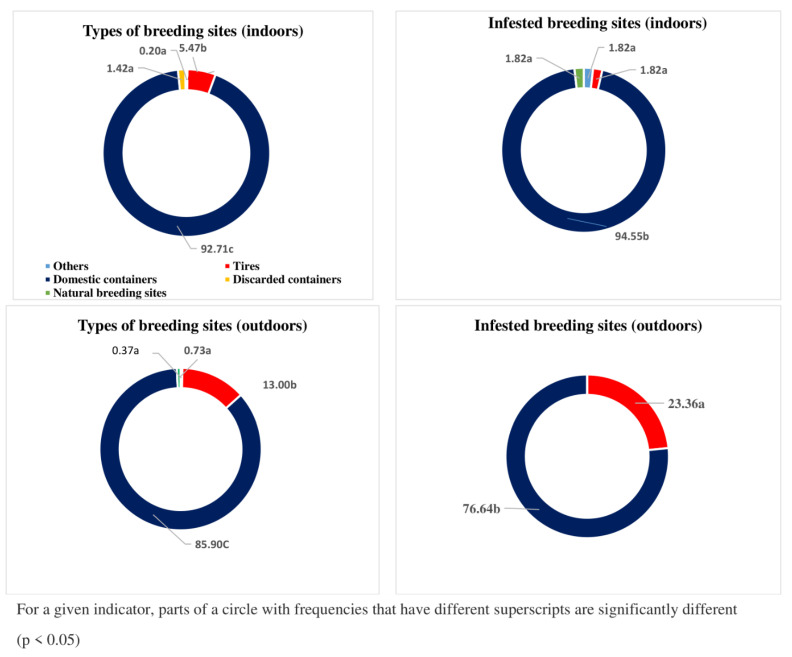
Proportion of the different types of breeding sites, as well as the positive sites inside and outside houses, in the study area.

In the study area, the predominant mosquito larval habitats that were encountered included domestic containers (92.71% indoors and 85.90% outdoors), followed by tires (5.47% indoors and 13% outdoors). Discarded containers (1.42% indoors and 0% outdoors), natural containers (0.20% indoors and 0.73% outdoors) and other types of breeding sites (0.20% indoors and 0.37% outdoors) were also found, but at very low frequencies (<2%) (Figure 3).

When indoors, the breeding sites infested with *Aedes* larvae were mostly (94.55%) domestic containers (water storage containers: cement-made cisterns, earthen jars, flowerpots, drums, cups, buckets, garbage cans, pet water bowls, bottles, mortars, barrels). When outdoors, three quarters of the positive *Aedes* breeding sites were also domestic containers (76.64%) (plastic bags, abandoned refrigerators and freezers, tin cans and coconuts) and the rest was composed of tires (23.36%).

## 4. Discussion

*Ae. albopictus*, a mosquito vector of arboviral diseases, has expanded its distribution area globally due to its ability to adapt to climate change and new environments [44,45,46]. The alarming increase in dengue fever cases in some West African countries in recent years has received relatively less attention in Benin. This situation is justified by the lack of information about the distribution of the main vectors that are involved. To the best of our knowledge, only one study confirmed the presence of *Ae. albopictus* in Benin [17]. This study provides an overview of the distribution and abundance of *Ae. aegypti* and *Ae. albopictus* in Benin and further identifies four other species of the *Aedes* genus.

The previous studies conducted in much smaller areas revealed the presence of four *Aedes* species in Cotonou [47] and three in Abomey-Calavi [21]. In the present study, *Ae. aegypti* was morphologically identified in all the surveyed communes with a relatively high frequency, ranging from 46.97% to 100%. This high abundance of *Ae. aegypti* could be justified by the native character of this species since its introduction [48]. The distribution pattern of *Ae. albopictus* is very recent [17]. Our findings supported the competitive superiority of *Ae. aegypti* to *Ae. albopictus* in all the ecological settings. These findings were in accordance with previous studies in Benin and Ghana [49,50]. This abundance of *Ae. aegypti* in Africa and particularly in Benin was significantly associated with unprecedented urbanization, poor management of solid waste, the movement of people and goods and, above all, climate change [51]. Numerous studies had already mentioned this cohabitation between the two species in Brazil [52], Malaysia [53] and neighboring Nigeria [54]. It has been reported that competition during larval development is shaping the distribution of both species [55]. Additionally, the previous studies suggested that the invasion of *Ae. albopictus* in most parts of the world has induced a decline in the abundance of *Ae. aegypti* over time and could even lead to its disappearance when both of them share the same larval breeding site due to the satyrisation effect [56]. However, this deserves further investigation and regular surveillance of the *Aedes* population dynamics. In our context in Benin, the conditions (ecological, climate or resources) were perhaps not yet sufficient for the rapid spread and dominance of *Ae. albopictus,* as previously reported in other African countries invaded by this species such as Cameroon, Côte d’Ivoire and Nigeria [57].

*Aedes aegypti* was found cohabiting with *Ae. albopictus* at 12/23 sites in this study. Given that Benin is located in the same eco-geographical region and shares approximately 773 km of border with Nigeria, it is, therefore, not surprising that the same trend was observed in certain communes of Benin that border Nigeria. This trend was further sustained by the fact that the two species shared the same ecological niches/larval habitats. Similarly, in Mayotte, *Ae. albopictus* coexisted with *Ae. aegypti* in 40% of the breeding sites [56]. This was also observed in the Southern communes of Benin that were surveyed in the present study. The broadly similar relative abundance of *Ae. albopictus* (51.72%) compared to the native species *Ae. aegypti* (46.97%) in Avrankou was not surprising since *Ae. albopictus* had been described as one of the most invasive [44]. In neighboring Nigeria, for example, *Ae. albopictus* was detected in 1991 [13]. Since then, the published data have shown that the species has become well established and has already exhibited dominance over *Ae. aegypti* in some parts of the country [58]. Moreover, both the rural and peri-urban characters of Avrankou could have favored the coexistence of *Ae. albopictus* and *Ae. aegypti* in this commune. According to Forattini et al. [59], Brady et al. [60] and Brown et al. [61], the urban environment could favor the proliferation of *Ae. aegypti*, while rural and suburban areas could be much more favorable to the development of *Ae. albopictus*. This hypothesis was supported by the work of Kamgang et al. [62] in Cameroon and the Central African Republic who showed that *Ae. albopictus* preferentially colonised containers that contained plant debris or were surrounded by vegetation; the types of larval habitats often found in rural areas.

In the study area, the *Aedes* larvae colonised mostly domestic breeding sites, both indoors and outdoors. This could have been due to poor water storage practices since water is generally stored for a long time in uncovered and sometimes unwashed jars, buckets and barrels for drinking, laundry, dishwashing and various construction activities. The water contained in tires abandoned outdoors also served as excellent breeding sites for *Aedes* mosquitoes. This showed the importance of the sensitization of the populations and the need to avoid both long periods of water storage and keeping anything outdoors that can retain water.

Our study assessed the distribution of *Aedes* mosquito species in different geographical areas in Benin. Although the invasive species *Ae. albopictus* has been recognised to colonise and adapt under different climate conditions, its distribution in Benin was restricted to the regions with a subequatorial climate. This was previously reported by Adeleke et al. [63] who also noticed that the mosquitoes rapidly spread in the southern part of neighboring Nigeria after its first detection in 1991.

However, it has been reported that the invasion of this mosquito in some countries started in coastal areas [64] and subsequently progressed inland [46]. The low altitude of the coastal region of Benin justified the high abundance of *Ae. albopictus* in the coastal communes compared to the communes in the center and the north of the country where it was not detected. According to Farjana et al. [65], the presence and the population density of these invasive species were highly dependent on climatic factors, such as the temperature, precipitation and relative humidity. No specimen of this species was collected under the Sudanian and Sudano-Guinean climates observed in northern part of Benin, which were more arid or semi-arid with relatively higher temperatures. The presence of *Ae. aegypti* and the absence of *Ae. albopictus* was associated with a better tolerance to desiccation by *Ae. aegypti* unlike *Ae. albopictus* [66]. Our findings were in accordance with the previous studies in Cameroon [67] and the Central African Republic [6]. However, it should be noted that the other studies reported the presence of *Ae. albopictus* in semi-arid and arid areas in Pakistan and Saudi Arabia, where dengue fever outbreaks have previously been reported [68,69]. In this context, the systematic sampling of mosquitoes conducted during longitudinal surveys covering both dry and rainy seasons with a good spatial coverage are essential for assessing the real distribution area of *Ae. albopictus,* particularly in arid areas.

The ovitraps collected the greatest number of *Aedes* spp. in this study. This result suggested that ovitraps were more attractive to gravid females and facilitated the sampling of *Aedes*. This confirmed the results of the previous studies that reported that, in many outbreaks, ovitraps showed a positivity for the presence of *Ae. aegypti* and *Ae. albopictus*, whereas with larval collection or HLC, lower numbers of these vectors were detected [70,71].

The molecular data confirmed the presence of *Ae. albopictus* in the twelve communes where it was morphologically identified. This result was similar to that obtained by Yadouleton et al. [17] who confirmed the presence of this species in Benin using molecular evidence.

The evaluation of the biting behavior of *Ae. aegypti* and *Ae. albopictus* performed in this study showed that these two vectors were mostly exophagic. This could be justified by the daytime biting behavior of *Aedes* mosquito species and the fact that the human hosts spent more time outdoors during the day. A similar trend was observed in Ghana [50] and Reunion Island. This exophagic behaviour of these main vectors could limit the effectiveness of vector control interventions that are often deployed inside homes, such as long-lasting insecticidal nets (LLINs) and indoor residual spraying (IRS). However, some studies conducted in Asia, Latin America [56,72], Côte d’Ivoire and Niger [73,74] revealed that *Ae. aegypti* was endophagic.

Although our study established the identification and distribution of *Ae. albopictus* in new localities in Benin, it did not focus on the diversity of the subspecies of *Ae. aegypti s.l*. This was a limitation that offered possibilities for future studies on *Ae. aegypti* subspecies in Benin. Another limitation worth noting was the sequencing of cytochrome oxidase 1 gene (COX1) in *Ae. albopictus*, which would have made it possible to establish phylogenetic links between this species found in southeastern Benin and the original species from Asia [75]. In addition, the Breteau, Container and House indices, which are commonly used to measure the risk incurred in terms of arbovirus transmission [76,77], could have been assessed to strengthen the present study.

## 5. Conclusions

This study provided the first data on the distribution, abundance and biting behaviour of the main arbovirus vectors *Ae. albopictus* and *Ae. aegypti* in Benin. It revealed that *Ae. albopictus* was well established in the southern part, but not in the northern part of Benin. Our findings supported the idea that arid climates prevailing in the northern part of Benin were not favorable for the establishment of the invasive species *Ae. albopictus*. A predominance of *Ae. aegypti* over *Ae. albopictus* was observed in all the investigated communes, with the exception of Avrankou, a southeastern commune of Benin bordering Nigeria. The study also reported that *Ae. aegypti* has become established in the domestic environment in Benin, with a high biting frequency of up to 4.02 bites/per person/day. The exophagic nature of these two vectors could limit the effectiveness of vector control interventions that are often implemented indoors (LLINs and IRS). The current study provided a map showing the distribution of *Ae. albopictus* and *Ae. aegypti* and will henceforth serve as a basis for the epidemiological surveillance of these vectors in Benin.

## Figures and Tables

**Table 1:** *Aedes* mosquito composition and abundance in the study sites between July 2021 and October 2022.

Localities (Communes)	*Ae. aegypti*	*Ae. albopictus*	*Ae. africanus*	*Ae. vitatus*	*Ae.* (*Neomelaniconion) palpalis*	*Ae. luteocephalus*	Total	Taxa (S)
	ni (Pi)	ni (Pi)	ni (Pi)	ni (Pi)	ni (Pi)	ni (Pi)	N	
Ifangni	1964 (47.83)	1731 (42.16)	0	0	1 (0.02)	410 (9.99)	4106	4
Kétou	139 (82.74)	22 (13.10)	1 (0.60)	0	3 (1.79)	3 (1.79)	168	5
Sakété	128 (55.41)	44 (19.05)	8 (3.46)	2 (0.87)	49 (21.21)	0	231	5
Pobè	771 (87.71)	92 (10.47)	0	0	0	16 (1.82)	879	3
Adja-Ouèrè	482 (87.96)	66 (12.04)	0	0	0	0	548	2
Porto-Novo	1653 (92.14)	133 (7.41)	0	0	0	4 (0.22)	1790	3
Adjara	128 (85.33)	22 (14.67)	0	0	0	0	150	2
Avrankou	178 (46.97)	196 (51.72)	0	2 (0.53)	3 (0.79)	0	379	4
Abomey–Calavi	181 (87.44)	26 (12.56)	0	0	0	0	207	2
Bohicon	252 (89.68)	29 (10.32)	0	0	0	0	281	2
Zangnanado	189 (96.92)	6 (3.08)	0	0	0	0	195	2
Lokossa	200 (100)	0	0	0	0	0	200	1
Klouekanmè	251 (100)	0	0	0	0	0	251	1
Grand-Popo	600 (100)	0	0	0	0	0	600	1
Djougou	241 (100)	0	0	0	0	0	241	1
Copargo	301 (100)	0	0	0	0	0	301	1
Savè	114 (100)	0	0	0	0	0	114	1
Parakou	314 (98.74)	0	0	4 (1.25)	0	0	318	2
Nikki	152 (98.70)	0	0	1 (0.65)	0	1 (0.65)	154	3
Bantè	551 (100)	0	0	0	0	0	551	1
Dassa	206 (86.19)	33 (13.81)	0	0	0	0	239	2
Ségbana	210 (99.52)	0	0	1 (0.47)	0	0	211	2
Gogounou	303 (97.74)	0	0	6 (1.93)	0	1 (0.32)	310	3
Total	9508	2400	9	16	56	435	12,424	-
Proportion (Pi) (%) [95% CI]	76.53 [75.77–77.27]	19.32 [18.63–20.02]	0.07 [0.03–0.14]	0.13 [0.07–0.21]	0.45 [0.34–0.58]	3.50 [3.18–3.84]	-	-

ni or N: number; Pi: proportion; CI: confidence interval.

**Table 2:** *Aedes* mosquito diversity indices in Benin.

Eco-Geographical Zones	Total	Taxas (S)	Equitability (J) [IC-95%]	Shannon (H) [IC-95%]
Subequatorial climate (Oueme, Plateau, Atlantic, Mono, Couffo and Zou regions)	9985	6	0.42 [0.41–0.43]	0.76 [0.74–0.77]
Sudanese-Guinean climate (Collines, Donga and Borgou regions)	1918	4	0.08 [0.05–0.10]	0.11 [0.08–0.14]
Sudanese climate (Alibori region)	521	3	0.07 [0.03–0.012]	0.08 [0.03–0.13]

**Table 3:** Distribution and abundance of *Aedes* species by climate type in Benin.

Eco-Climatic Zones	*Ae. aegypti*	*Ae. albopictus*	*Ae. luteocephalus*	*Others Aedes*	Total
	N (%)	N (%)	N (%)	N (%)	
Subequatorial climate (Oueme, Plateau, Atlantic, Mono, Couffo and Zou regions)	7116 (71.27)	2367 (23.71)	433 (4.34)	69 (4.34)	9985
Sudanese-Guinean climate (Collines, Donga and Borgou regions)	1879 (97.97)	33 (1.72)	1 (0.05)	5 (0.26)	1918
Sudanese climate (Alibori region)	513 (98.46)	0	1 (0.19)	7 (1.34)	521

**Table 4:** Abundance and diversity of *Aedes* species using the sampling method in Benin.

Climates and *Aedes* Species	Human Landing Catch (HLC)	Larvae and Pupae Collection	Ovitrap Method	Total
	n (%)	n (%)	n (%)	N
**Subequatorial climate**				
*Ae. aegypti*	1398 (19.65)	151 (2.12)	5567 (78.23)	7116
*Ae. albopictus*	131 (5.53)	54 (2.28)	2182 (92.18)	2367
*Ae. luteocephalus*	4 (0.92)	0 (0.00)	429 (99.08)	433
*Ae. palpalis*	56 (100.00)	0 (0.00)	0 (0.00)	56
*Ae. africanus*	3 (33.33)	6 (66.67)	0 (0.00)	9
*Ae. vitatus*	4 (100.00)	0 (0.00)	0 (0.00)	4
**Sudano-Guinean climate**				
*Ae. aegypti*	1480 (78.77)	348 (18.52)	51 (2.71)	1879
*Ae. albopictus*	33 (100.00)	0 (0.00)	0 (0.00)	33
*Ae. vitatus*	4 (80.00)	0 (0.00)	1 (20.00)	5
*Ae. luteocephalus*	0 (0.00)	1 (100.00)	0 (0.00)	1
**Sudanian climate**				
*Ae. aegypti*	253 (49.32)	197 (38.40)	63 (12.28)	513
*Ae. vitatus*	2 (28.57)	5 (71.43)	0 (0.00)	7
*Ae. luteocephalus*	0 (0.00)	0 (0.00)	1 (100.00)	1
**All areas**				
*Ae. aegypti*	3131 (32.93)	688 (7.32)	5681 (59.75)	9508
*Ae. albopictus*	164 (6.83)	54 (2.25)	2182 (90.92)	2400
*Ae. luteocephalus*	4 (0.92)	1 (0.23)	430 (98.85)	435
*Ae. palpalis*	56 (100.00)	0 (0.00)	0 (0.00)	56
*Ae. africanus*	3 (33.33)	6 (66.67)	0 (0.00)	9
*Ae. vitatus*	10 (62.50)	5 (31.25)	1 (6.25)	16
Total	3368 (27.11)	762 (6.13)	8294 (66.75)	12,424

*Ae: Aedes.*

**Table 5:** Molecular identification of *Ae. albopictus* in Benin.

Localities (Communes)	N-Tested	N-Confirmed
Abomey-Calavi	26	26
Adjarra	22	22
Avrankou	30	30
Ifangni	30	30
Kétou	22	22
Porto-Novo	30	30
Bohicon	29	29
Adja-ouèrè	30	30
Dassa	30	30
Pobè	30	30
Zangnanado	6	6
Sakété	30	30
Total	315	315

N: number.

**Table 6:** HBR in *Ae. aegypti* between July 2021 and October 2022.

Localities (Communes)	Periods	Nb *Ae. aegypti* Collected		HBR/Day					CI-95%
		Indoors	Outdoors	Total	Indoors	Outdoors	RR and CI-95%	Total HBR /Day	
Porto-Novo	May 2021	41	70	111	5.13 ^a^	8.75 ^b^	0.58 [0.38–0.87]	6.94	[5.71–8.35]
	July 2021	5	145	150	0.63 ^a^	18.13 ^b^	0.03 [0.01–0.08]	9.38	[7.93–11.00]
	October 2022	9	22	31	1.13 ^a^	2.75 ^b^	0.4 [0.16–0.92]	1.94	[1.32–2.75]
**Total**	Rainy season (May 2021–October 2022)	55	237	292	2.29 ^a^	9.88 ^b^	0.23 [0.17–0.31]	6.08	[5.41–6.82]
Ifangni	May 2021	7	49	56	0.88 ^a^	6.13 ^b^	0.14 [0.05–0.32]	3.5	[2.64–4.54]
	July 2021	13	40	53	1.63 ^a^	5 ^b^	0.32 [0.16–0.62]	3.31	[2.48–4.33]
**Total**	Rainy season (May–July 2021)	20	89	109	1.25 ^a^	5.56 ^b^	0.22 [0.13–0.37]	3.41	[2.80–4.11]
Kétou	Dry season (August 2022)	2	20	22	0.25 ^a^	2.5 ^b^	0.1 [0.01–0.41]	1.38	[0.86–2.08]
Avrankou	Dry season (August 2022)	1	2	3	0.13 ^a^	0.25 ^a^	0.5 [0.00–9.6]	0.19	[0.038–0.55]
**Overall total**	Dry and rainy seasons (May 2021–August 2022)	153	674	827	1.59 ^a^	7.02 ^b^	0.23 [0.19–0.27]	4.31	[4.02–4.61]

Nb: number; HBR: human biting rate; CI: confidence intervals; RR: rate ratio; HBR of *Ae. aegypti* with different superscript (a, b) are significantly different (*p* < 0.05).

**Table 7:** HBR of *Ae. albopictus* between July 2021 and October 2022.

Localities (Communes)	Periods	Nb *Ae. albopictus* Collected			HBR/Day				CI-95%
		Indoors	Outdoors	Total	Indoors	Outdoors	RR and CI-95%	Total HBR /Day	
Porto-Novo	May 2021	2	5	7	0.25 ^a^	0.63 ^a^	0.4 [0.04–2.44]	0.44	[0.17–0.90]
	July 2021	1	5	6	0.13 ^a^	0.63 ^b^	0.2 [0.004–1.79]	0.38	[0.14–0.82]
	October 2022	2	13	15	0.25 ^a^	1.63 ^a^	0.15 [0.02–0.68]	0.94	[0.52–1.54]
**Total**	Rainy season (May 2021–October 2022)	5	23	28	0.21 ^a^	0.96 ^b^	0.22 [0.06–0.58]	0.58	[0.39–0.84]
Ifangni	May 2021	6	15	21	0.75 ^a^	1.87 ^a^	0.4 [0.13–1.09]	1.31	[0.81–2.00]
	July 2021	5	9	14	0.63 ^a^	1.13 ^a^	0.55 [0.15–1.84]	0.88	[0.48–1.47]
**Total**	Rainy season (May–July 2021)	11	24	35	0.69 ^a^	1.50 ^b^	0.46 [0.20–0.97]	1.09	[0.76–1.52]
Ketou	Dry season (August 2022)	0	12	12	0.00 ^a^	1.50 ^b^	0 [0.00–0.36]	0.75	[0.39–1.31]
Avrankou	Dry season (August 2022)	5	39	44	0.63 ^a^	4.88 ^b^	0.13 [0.04–0.32]	2.75	[2.00–3.69]
**Overall total**	Dry and rainy seasons (May 2021–August 2022)	37	145	182	0.39 ^a^	1.51 ^b^	0.25 [0.17–0.37]	0.95	[0.81–1.09]

Nb: number; HBR: human biting rate; CI: confidence intervals; RR: rate ratio; HBR of *Ae. albopictus* with different superscript (a, b) are significantly different (*p* < 0.05).

## Data Availability

The datasets that were analysed in this study are available from the corresponding authors upon reasonable request.
